# Genetic Variants in *STAT3* Promoter Regions and Their Application in Molecular Breeding for Body Size Traits in Qinchuan Cattle

**DOI:** 10.3390/ijms19041035

**Published:** 2018-03-29

**Authors:** Sen Wu, Yaning Wang, Yue Ning, Hongfang Guo, Xiaoyu Wang, Le Zhang, Rajwali Khan, Gong Cheng, Hongbao Wang, Linsen Zan

**Affiliations:** 1 College of Animal Science and Technology, Northwest A & F University, Yangling 712100, China; wusenkgdsss@hotmail.com (S.W.); wangyn1992@outlook.com (Y.W.); ningyue@nwsuaf.edu.cn (Y.N.); guohongfang@nwsuaf.edu.cn (H.G.); wangxiaoyu@nwsuaf.edu.cn (X.W.); zhangle@nwsuaf.edu.cn (L.Z.); rajwalikhan@aup.edu.pk (R.K.); cg4455@gmail.com (G.C.); wanghongbao@nwsuaf.edu.cn (H.W.); 2 National Beef Cattle Improvement Center of Northwest A & F University, Yangling 712100, China

**Keywords:** STAT3, promoter SNP, mRNA expression, conserved estimation, body size traits, molecular markers

## Abstract

Signal transducer and activator of transcription 3 (STAT3) plays a critical role in leptin-mediated regulation of energy metabolism. This study investigated genetic variation in *STAT3* promoter regions and verified their contribution to bovine body size traits. We first estimated the degree of conservation in STAT3, followed by measurements of its mRNA expression during fetal and adult stages of Qinchuan cattle. We then sequenced the *STAT3* promoter region to determine genetic variants and evaluate their association with body size traits. From fetus to adult, STAT3 expression increased significantly in muscle, fat, heart, liver, and spleen tissues (*p* < 0.01), but decreased in the intestine, lung, and rumen (*p* < 0.01). We identified and named five single nucleotide polymorphisms (SNPs): SNP1-304A>C, SNP2-285G>A, SNP3-209A>C, SNP4-203A>G, and SNP5-188T>C. These five mutations fell significantly outside the Hardy–Weinberg equilibrium (HWE) (Chi-squared test, *p* < 0.05) and significantly associated with body size traits (*p* < 0.05). Individuals with haplotype H3H3 (CC-GG-CC-GG-CC) were larger in body size than other haplotypes. Therefore, variations in the *STAT3* gene promoter regions, most notably haplotype H3H3, may benefit marker-assisted breeding of Qinchuan cattle.

## 1. Introduction

Body size is a pleiotropic suite of traits essential to livestock genetic breeding programs. Successfully applying marker-assisted selection (MAS) in livestock depends on the identification of relevant genes or tightly linked markers [1]. Growth rate is one aspect of body size that can be selected for based on candidate gene associations. This method is more straightforward than other genomic approaches, suggesting it can be implemented efficiently and accurately in breeding programs. Candidate genes or QTL (quantitative trait loci) are identified via testing for relationships between physiological or biochemical characteristics linked to body size traits [2]. Thus far, promising candidate genes for improving body size traits include *SIRT2*, *MTNR1A*, *SIX4*, *MC4R* and *FTO*, identified in cattle, pigs, and other livestock [3,4,5,6].

Signal transducer and activator of transcription 3 (STAT3) is a ubiquitous cytoplasmic protein expressed in multiple metabolic tissues. It is a member of the STAT protein family, characterized by the presence of Src homology domain 2 (SH2), Src homology domain 3 (SH3), and a tyrosine phosphorylation site at the carboxyl-terminal region. Various cytokines and growth factors phosphorylate STAT3 and translocate the activated protein to the cell nucleus, where it functions as a transcription factor [7,8]. Acetylation, deacetylation, and dephosphorylation of STAT3 results in metabolism disturbance and occasionally disease [9,10,11,12]. In the hypothalamus, STAT3 is critical to leptin-mediated regulation of energy metabolism [13], as evidenced by the fact that its deletion interferes with normal body weight homeostasis and glucose metabolism [14]. Moreover, knockout mouse studies have shown that disrupting neural STAT3 causes leptin-resistant conditions such as obesity, diabetes, and thermal dysregulation [15]. Mice with pancreatic beta-cell-specific disruption of the *STAT3* gene exhibited increased appetites, obesity, partial leptin resistance, and glucose intolerance [16]. Interestingly, a recent mouse study showed that *STAT3* regulates differentiation of brown adipose tissues (BAT), involved primarily in burning energy [17]. Genetic variants of *STAT3* in cattle directly influence body size and carcass quality traits [18].

Qinchuan cattle have been popular farming and meat breeds in China for thousands of years [19], due to its tall body, genetic stability, and adaptability. However, when faced with competition from foreign commercial beef cattle, Qinchuan’s economic benefits are severely curtailed by shortcomings such as underdeveloped hindquarters and slow growth rate. Cattle body-size traits are difficult to improve using traditional breeding methods. However, the candidate gene approach may be useful for uncovering associations between gene polymorphisms and economically valuable traits in farm animals [20]. Previous research has identified many genes related to growth [21], production [22], and meat quality [23].

Because STAT3 is important to almost every aspect of energy metabolism, its variants should predispose carriers to specific body-size traits. To the best of our knowledge, few studies have investigated this possibility in cattle, with most research focusing on humans and laboratory animals [24,25,26,27]. Genetic variations in promoter regions are also extremely relevant to economically valuable traits in livestock, due to their quantitative impact on gene expression [28,29]. For example, SNPs in the *SIRT3* promoter region influences intramuscular fat deposition in beef cattle [30], while SNPs in the *GPAT3* promoter region are associated with pig body-size traits and promoter activity [31], and so on. Therefore, variation in bovine *STAT3* promoter regions should be investigated and their contribution to Qinchuan body size verified.

In the present study, we used bioinformatics techniques to predict *STAT3* gene function. We then detected *STAT3* mRNA expression at the fetal and adult stages of Qinchuan cattle. Next, we sequenced the *STAT3* promoter region in 420 Qinchuan cattle to analyzed genetic variation. Finally, we tested for associations between SNPs and haplotype combinations with body size traits of Qinchuan cattle. Our results should greatly benefit MAS breeding programs.

## 2. Results

### 2.1. Biological Evolution and Estimates of Conservation

**Figure 1:**
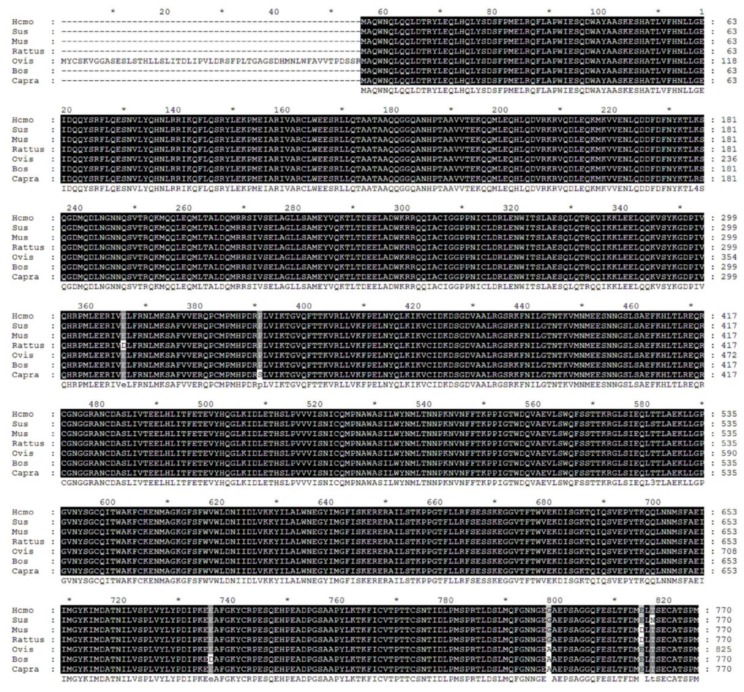
Multiple sequence alignment of STAT3 protein across seven species. The degree of similarity is delineated using different background shading, with black being 100%; grey with black text, 80%; grey with white text, 60%; and white, not conserved.

**Figure 2:**
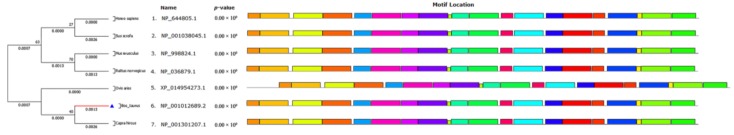
Phylogenetic tree (**Left**) and Motif structural analysis (**Right**) for seven species. Twenty significant motifs were identified. The length of the color block shows the position, strength and significance of a particular motif site. The length of the motif is proportional to the negative logarithm of the *p*-value of the motif site, truncated at the height for a *p*-value of 1 × 10^−10^. These colors are given through motif analysis performed through MEME suit system. The “red line” and “blue triangle” are specifically conserved motif sites for *Bos Taurus* species.

**Figure 3:**
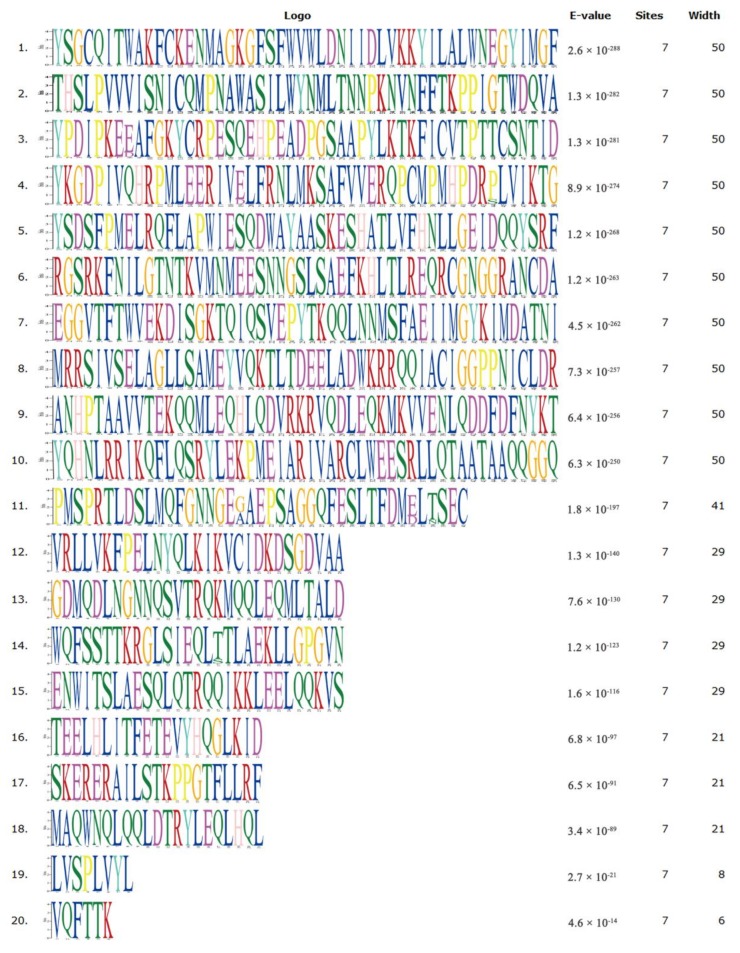
Significant STAT3 motifs across the seven tested species, detected using the MEME suite. The different color letters show abbreviation of different amino acids. These colors are given through motif analysis performed through MEME suit system.

**Figure 4:**
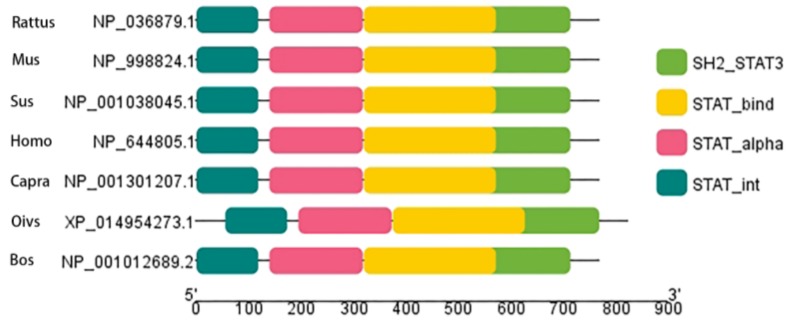
Structure of STAT3 protein domain families in seven species. Each color block is a specific hit representing a different domain superfamily.

We performed multiple sequence alignment on STAT3 from seven species, including common ruminants (bovine, goat, and sheep), monogastric animals (rat, mouse, pig), and humans (Figure 1). As the primary structure was highly similar among species, we hypothesized that function was also similar across tested species. We then investigated STAT3 sequence phylogenetic tree construction (Figure 2) and used the MEME suite tool to look for common motifs in supersecondary structures (Figure 2 and Figure 3). Bovine, goat, and sheep STAT3 were the most closely related, while human, pig, rat, and mouse branches were far from the bovine sequence. We found 20 significant motifs among the seven species (Figure 2 and Figure 3), indicating functional similarity at the supersecondary structure level. We searched NCBI CDD for protein tertiary structures and found the same four specific hits per species, representing four domain superfamilies (Figure 4). Thus, each species possesses a STAT3 protein with four similar domain structures (SH2_STAT3, STAT_bind, STAT_alpha, and STAT_int superfamily), at the tertiary level, providing further evidence of functional similarity.

### 2.2. Differential Expression of STAT3 across Development

**Figure 5:**
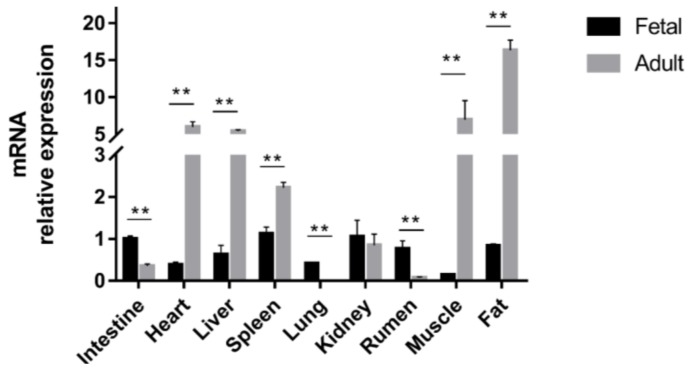
Differential *STAT3* mRNA expression across tissues of Qinchuan cattle (*n* = 3). Double asterisks (**) indicate significant differences between fetal and adult stages (*p* < 0.01).

We determined *STAT3* mRNA expression in nine different tissues from fetal (FQC) and adult (AQC) Qinchuan cattle. Relative expression varied in all examined tissues (Figure 5). Among FQC, STAT3 expression was highest in fat, rumen, kidney, spleen, and intestine; moderate in lung, liver, and heart; and lowest in muscle. However, among AQC, STAT3 expression was highest in fat, muscle, heart, and liver; moderate in spleen; and lowest in intestine, lung, kidney, and rumen.

We observed a significant increase in STAT3 expression of heart, liver, spleen, muscle, and fat from the fetal to adult stage (*p* < 0.01). In contrast, intestinal, lung, and ruminal STAT3 expression decreased dramatically among AQC (*p* < 0.01). Finally, renal STAT3 expression did not differ between FQC and AQC.

These results suggest either a direct or indirect connection between bovine body size traits and STAT3 action, considering that the protein’s biological processes are highly conserved across mammals. Overall, STAT3 regulatory function warrants further research.

### 2.3. Identification of Sequence Variants and Prediction of Transcription Factors in Qinchuan Cattle

**Figure 6:**
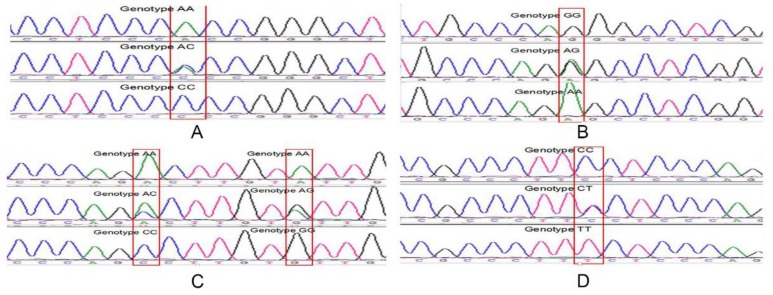
Sequence variants of the *STAT3* promoter. Annotations A, B, C, and D represent the five SNP sites and the resultant genotypes.

We identified five SNPs in STAT3 promoter regions: SNP1-304A>C, SNP2-285G>A, SNP3-209A>C, SNP4-203A>G, and SNP5-188T>C. Sequencing each SNP respectively yielded genotypes AA, AC, CC; GG, AG, AA; AA, AC, CC; GG, AG, AA; and TT, CT, CC (Figure 6). Genotypes and allele frequencies were analyzed for the five mutations (Table 1), which were found to be significantly outside HWE (Chi-squared test, *p* < 0.05). Additionally, PIC classification indicated that the five SNPs were moderately polymorphic (0.25 < PIC < 0.50).

In silico analysis indicated that alternative alleles may generate gains or losses of transcription factor binding sites. The substitution of A with C in SNP1 and SNP3, as well as the substitution of T with C in SNP 5 produced a putative gain of binding sites Sp1, MyoD, and SRF, respectively (Table 2). Substituting G with A in SNP2 and A with G in SNP4 produced a putative loss of binding sites AP-2 and CP1, respectively.

### 2.4. Linkage Disequilibrum (LD) and Haplotype Analysis

The most commonly used predictors of LD are D′ and *r*^2^. The latter index is a pairwise measure of LD and less sensitive to allele frequencies than D’ [32,33]. We found that D′ ranged from 0.235 to 1.000 among the five SNPs, while *r*^2^ range was 0.052–0.452 (Table 3). When *r*^2^ > 0.33, LD is considered to be sufficiently strong for use in mapping [34]. Based on both predictors, LD was strongest between SNP3 and SNP4.

Haplotype analyses were performed using the online tool SHEsis. Because we were interested in common genetic polymorphisms (frequency ≥0.05) [35], all haplotypes with frequencies <0.05 were excluded, leaving Hap1 (AAAAT), Hap2 (CGAAC), and Hap3 (CGCGC) (Table 4). The third haplotype was the most frequently occurring High-frequency haplotypes are probably ancient and better adapted to the current environment [36]. Most new mutants were derived from common haplotypes, implying that rare variants represented recent mutations and were likely related to common haplotypes [37].

### 2.5. Effects of SNPs and Haplotype Combinations on Body Size Traits

We examined relationships between the five SNPs and body size traits in 420 Qinchuan cattle (Table 5). At the SNP1 locus, genotype-AA individuals had greater chest depth than AC and CC individuals (*p* < 0.05). However, body length, wither height, hip height, hip width, and chest circumference did not differ between genotypes (*p* > 0.05). At the SNP2 locus, AG and AA individuals had greater wither height than GG individuals (*p* < 0.01). At the SNP3/4 locus, genotypes AA/AA and AC/AG were significantly related to wither height and chest depth (*p* < 0.01). Additionally, chest depth differed significantly between these two genotypes (*p* < 0.05), whereas they were not associated with other body-size parameters (*p* > 0.05). At the SNP5 locus, TT individuals had greater hip width, hip height (*p* < 0.01) and chest depth (*p* < 0.05) than CC individuals. Table 6 lists the associations of haplotype combinations with body size traits. Combinations of frequencies <5.0% were excluded from further analysis. The haplotype combination H3H3 (CC-GG-CC-AA-CC) yielded significantly enhanced body size traits than other combinations (*p* < 0.01).

## 3. Discussion

The bovine *STAT3* gene (24 exons) is located on chromosome 19 and is involved in leptin-mediated regulation of energy metabolism [13,14,15,16,17]. The leptin receptor–STAT3 signaling pathway is central to leptin regulation of food intake and energy expenditure [38]. In vitro studies reported that *STAT3* deletion interferes with normal body weight homeostasis and glucose metabolism, leading to obesity, diabetes, and thermal dysregulation [14,15]. Furthermore, STAT3 loss in mature adipocytes of mice increased adiposity and adipocyte hypertrophy [39]. Among livestock, *STAT3* polymorphisms significantly affected body size traits in Xinong Saanen dairy goats and Hainan black goats [40]. Identification of candidate genes and QTLs is useful for marker-assisted breeding to improve economically important traits in cattle. Numerous findings [14,15,38,39] suggest that STAT3 influences muscle and adipose tissue growth. Unfortunately, few studies have examined how bovine *STAT3* promoter regions are associated with body size traits in Qinchuan cattle. Thus, here we successfully identified five SNPs in bovine *STAT3* promoter regions. Chi-squared tests revealed that the five SNPs were not in HWE, possibly due to artificial selection from draft cattle to beef production, or small sample size [41].

Promoters can alter QTL expression through regulating mRNA isoforms [42]. In the present study, tissue-preferential STAT3 expression differed significantly across fetal and adult stages. Moreover, we found significant relationships between SNPs and body size traits in our cattle population. Specifically, SNP1 genotype AA influenced chest depth (*p* < 0.05), while SNP2 genotypes AG and AA were associated with improved wither height (*p* < 0.01). The SNP3/4 genotypes AA/GG and AC/AG also affected wither height and chest depth (*p* < 0.05), while genotype CC/AA resulted in the widest hips (*p* < 0.01). At SNP5, genotype TT increased hip height, hip width, wither height, and chest depth over genotype CC (*p* < 0.05). Our investigation of associations between haplotype combinations and body size traits revealed that H3H3 individuals differed significantly from other haplotypes (*p* < 0.01). These results suggested that H3H3 could see potential use as a molecular marker in future breeding programs to increase Qinchuan cattle growth rates. In our future studies, we aim to investigate how SNPs alter *STAT3* promoter activity to influence body size traits.

In complex signaling cascades, transcription factors activate the target gene via directly binding to DNA, or else control gene expression through altering chromatin configuration [43]. Thus, we used allelic presence to predict potential transcription factor binding sites. Mutations in SNP1, SNP3, and SNP5 produced a putative gain of Sp1, MyoD, and SRF binding sites, respectively, whereas mutations in SNP2 and SNP4 produced a putative loss of AP-2 and CP1 binding sites. This outcome suggests that the identified SNPs would affect transcription-factor binding affinity in surrounding sequences. However, gene expression studies should be performed to confirm exact SNP function.

Methylation of *STAT3* can influence relevant traits through altering gene activity, leading to consequences such as increasing the risk of gastric cancer [44]. In turn, variants of a given promoter region may alter methylation. Previous studies have shown that STAT3 activation causes changes to colorectal cancer [45], glioma cells [46], and dendritic cells [47]. In this study, we found clear associations between *STAT3* promoter SNPs and body size traits of Qinchuan cattle. The underlying mechanism of this link may be related to methylation-induced promoter activation. However, further research is necessary before we can fully understand how promoter methylation influences the association between *STAT3* and traits of interest.

## 4. Materials and Methods

### 4.1. Bioinformatics Analyses

Amino acid sequences of STAT3 were acquired from NCBI for seven species (*Bos taurus* NP_001012689.2, *Homo sapiens* NP_644805.1, *Rattus norvegicus* NP_036879.1, *Mus musculus* NP_998824.1, *Capra hircus* NP_001301207.1, *Ovis aries* XP_014954273.1, and *Sus scrofa* NP_001038045.1). Multiple sequence alignment was performed in MUSCLE (MUltiple Sequence Comparison by Log-Expectation), while a neighbor-joining phylogenetic tree was constructed in MEGA version 7.0.26 (Philadelphia, PA, USA) [48]. To analyze protein-structure function, we searched motifs and conserved domains using the MEME suite [49] and NCBI CDD [50,51].

### 4.2. Subject Animals

The Experiment Farm of the National Beef Cattle Improvement Center (Yangling, China) supplied Qinchuan cattle for this study. The experiment used 420 adult females (aged 24–30 months, unrelated for at least three generations, not pregnant). All procedures were performed in accordance with the guidelines of the China Council on Animal Care. Protocols were also approved by the Experimental Animal Management Committee (EAMC) of Northwest A & F University (EAMC.N0.2013-23, 20 April 2013). The same care protocol and the same environment were employed for cattle rearing. Subjects were fed a diet of 25% concentrate and 75% roughage (corn silage and dry straw) on a total mixed ration (TMR) basis and provided water *ad libitum*, following based on the Nutrient Requirement of Beef Cattle (Eighth Revised Edition, NRC, 2016).

### 4.3. RNA Preparation and Real-Time PCR

Three fetal Qinchuan cattle (FQC, 90-day-old) and three adult Qinchuan cattle (AQC, 24-month-old) were randomly selected (three biological replicates per age). The adults and fetuses were unrelated within the last three generations. The FQC embryos (cattle gestation: 280 days) were placed in sterile physiological saline immediately after removal from the reproductive tract of slaughtered cattle at a local abattoir. In addition, FQC age was estimated following published research [52].

To detect transcriptome-level STAT3 expression, we collected nine tissues (intestine, heart, liver, spleen, lung, kidney, rumen, muscle, and fat) at both FQC and AQC. Samples were immediately frozen in liquid nitrogen upon collection from the carcasses before being transferred to the laboratory for RNA extraction.

The RNAprep Pure Tissue kit (Tiangen, Beijing, China) and reverse transcription kit (Thermo Fisher Scientific, Waltham, MA, USA) were used for RNA extraction and cDNA synthesis, respectively. Real-time quantitative PCR (RT-qPCR) was performed in an Applied Biosystems thermocycler (ABI7500, Thermo Fisher Scientific, Waltham, MA, USA), using the SYBR Premix Ex Taq kit (Takara, Dalian, China). Bovine *β-actin* and *GAPDH* were used as internal controls. Table 7 provides all primers used. Analyses were performed in triplicate. Relative expression of mRNA was calculated using the 2^−ΔΔ*C*t^ method [53].

### 4.4. DNA Isolation, and Phenotypic Data

Blood samples for DNA extraction were collected from 420 Qinchuan cattle, aged 24–30 months, following published protocols [54]. Body size traits (body length, withers height, hip height, rump length, hip width, chest depth, and chest circumference) were measured in accordance with previous methods [55].

### 4.5. PCR Amplification and Genotyping

Promoter regions were PCR-amplified (primers: F-GGAACGAAGGGCAGGGTTAAA; R-GCTGGGGTGCTCGTCAGGGAT) sequenced. The reaction mixture (20 μL) contained 50 ng DNA, 10 pM of each primer, 0.20 mM dNTP, 2.5 mM MgCl_2_, and 0.5 U Taq DNA polymerase (Takara, Dalian, China). Amplicons were sequenced in Sangon (Shanghai, China) to screen for variants. Thermocycling conditions were as follows: 95 °C for 5 min; 94 °C for 30 s, 63.1 °C for 35 s, and 72 °C for 40 s; 35 cycles from 94 °C to 72 °C; followed by a final extension at 72 °C for 10 min. Sequences were identified in SeqMan (DNASTAR, Inc., Madison, WI, USA). Promoter positions were numbered by designating the first nucleotide of the first exon as +1 and the nucleotide immediately upstream as −1.

### 4.6. Data Analyses

Genotypic and allelic frequencies were directly calculated for all five SNPs. The Hardy-Weinberg equilibrium (HWE) was estimated with a Chi-squared test in PopGene version 3.2 (University of Alberta, Edmonton, AB, Canada) [56]. Population genetic indices, including gene heterozygosity (He) and polymorphism information content (PIC), were statistically analyzed following published methods [57]. General linear models were used to analyze trait means. The relationship between different genotypes and body size traits of Qinchuan cattle was analyzed in SPSS 24.0 (SPSS, Inc., Chicago, IL, USA). The statistical linear model for this analysis was the same as previous reports [58,59]: Yijk = u + Gi + Ai + Sk + Eijk, with Yijk = trait value per individual, μ = overall population mean per trait, Gi = fixed effect associated with genotype, Ai = fixed effect of age, and Eijk = standard error. Putative binding sites for transcription factors were searched using TESS (available online: http://gene-regulation.com/pub/databases.html).

Linkage disequilibrium (LD) and haplotypes were analyzed using SHEsis [60]. The Bonferroni correction was used to adjust *p* values.

## 5. Conclusions

In this study, we determined that the combined genotype H3H3 (CC-GG-CC-AA-CC) had the strongest effect on body size traits among all identified SNP variants of *STAT3* promoter regions. We conclude that this genotype could be used as a molecular marker in future breeding programs that aim to select for body size traits in Qinchuan cattle.

## Figures and Tables

**Table 1:** Genotypes, Minor Allele Frequency, Hardy-Weinberg equilibrium (HWE), He, Ne, and PIC for the five SNPs.

Loci	Genotype Frequency			Allele Frequency		HWE	PIC	He	Ne
SNP1	AA	AC	CC	A	C				
	0.2405	0.1833	0.5762	0.3321	0.6679	144.6004	0.3452	0.4436	1.7974
SNP2	AA	AG	GG	A	G				
	0.2857	0.1429	0.5714	0.3571	0.6429	199.3185	0.3538	0.4592	1.8491
SNP3/4	AA/GG	AC/AG	CC/AA	A/G	C/A				
	0.2143	0.5952	0.1905	0.5119	0.4881	15.3463	0.3749	0.4997	1.9989
SNP5	CC	CT	TT	C	T				
	0.5238	0.3095	0.1667	0.6786	0.3214	35.4313	0.3411	0.4362	1.7738

Note: SNP1: −304A>C, SNP2: −285G>A, SNP3: −209A>C, SNP4: −203A>G, SNP5: −188T>C; χ_0.05_^2^ = 5.991, χ_0.01_^2^ = 9.21; He: gene heterozygosity; Ne: effective allele numbers; PIC: polymorphism information content.

**Table 2:** In silico alteration of nucleotides to generate alternative SNP alleles, resulting in putative gain or loss of transcription factor binding sites.

SNPs	Variation	Predicted Transcription Factor Binding Site			
		Gain ^a^	Loss ^b^	Transcription Factor Binding Sequence ^c^	Score
SNP1	−304A>C	-	Sp1	CCCCC	100
SNP2	−285G>A	AP-2	-	CCCAGGG	100
SNP3	−209A>C	-	MyoD	CAGCC	100
SNP4	−203A>G	CP1	-	ATTGC	100
SNP5	−188T>C	-	SRF	TTCCTC	100

Note: ^a,b^ Generated after substitution of allele 1 (wild type) with allele 2 (mutant); ^c^ Based on TFSEARCH analysis.

**Table 3:** Estimated values of linkage equilibrium between five SNPs in Qinchuan cattle.

SNP	QC	
	*r*^2^	D′
SNP1-SNP2	0.211	0.486
SNP1-SNP3	0.072	0.391
SNP1-SNP4	0.060	0.340
SNP1-SNP5	0.052	0.235
SNP2-SNP3	0.149	0.530
SNP2-SNP4	0.091	0.369
SNP2-SNP4	0.276	0.569
SNP3-SNP4	0.452	1.000
SNP4-SNP5	0.332	0.817

**Table 4:** Haplotypes and their frequencies in Qinchuan cattle (frequency >0.05 are shown).

Haplotypes	SNP1	SNP2	SNP3	SNP4	SNP5	Frequencies
Hap1	A	A	A	A	T	0.151
Hap2	C	G	A	A	C	0.112
Hap3	C	G	C	G	C	0.332

**Table 5:** Association of different SNP genotypes with body size traits in Qinchuan cattle.

Loci	Genotypes	Body Size Traits (Mean ± SD)					
		Body Length (cm)	Wither Height (cm)	Hip Height (cm)	Hip Width (cm)	Chest Depth (cm)	Chest Circumference (cm)
SNP1	AA	127.683 ± 1.051	117.990 ± 0.623	119.119 ± 0.434	34.297 ± 0.617	59.446 ± 0.729 ^a^	155.574 ± 1.775
	AC	127.753 ± 1.203	117.325 ± 0.714	119.636 ± 0.497	34.260 ± 0.707	57.961 ± 0.835	152.740 ± 2.033
	CC	127.839 ± .679	116.855 ± 0.403	119.326 ± 0.280	34.083 ± 0.399	57.430 ± 0.471 ^b^	154.694 ± 1.147
SNP2	AA	127.875 ± 0.969	118.567 ± 0.564 ^A^	119.667 ± 0.419	34.833 ± 0.566	58.167 ± 0.672	155.750 ± 1.641
	AG	128.667 ± 1.370	118.683 ± 0.798 ^A^	119.583 ± 0.593	33.333 ± 0.801	59.333 ± 0.951	154.833 ± 2.321
	GG	127.146 ± 0.685	115.833 ± 0.399 ^B^	118.500 ± 0.297	33.917 ± 0.400	57.479 ± 0.475	153.583 ± 1.161
SNP3/4	AA/AA	129.056 ± 1.100	119.278 ± 0.656 ^A,a^	120.000 ± 0.482	34.778 ± 0.645	60.222 ± 0.765 ^A^	157.556 ± 1.876 ^a^
	AC/AG	125.984 ± 0.661	116.279 ± 0.394 ^B^	118.681 ± 0.290	33.209 ± 0.388 ^B^	56.787 ± 0.460 ^B^	152.185 ± 1.128 ^b^
	CC/GG	130.938 ± 1.167	116.938 ± 0.696 ^b^	118.938 ± 0.511	36.125 ± 0.684 ^A^	59.000 ± 0.811	157.875 ± 1.990 ^a^
SNP5	TT	128.143 ± 1.269	118.071 ± 0.748 ^a^	119.962 ± 0.400 ^A^	35.273 ± 0.411 ^A^	59.714 ± 0.873 ^a^	156.000 ± 1.575
	TC	127.885 ± 0.931	118.038 ± 0.549	119.500 ± 0.545	33.429 ± 0.728	58.692 ± 0.641	155.143 ± 2.147
	CC	127.205 ± 0.716	116.114 ± 0.422 ^b^	118.250 ± 0.307 ^B^	32.462 ± 0.534 ^B^	56.932 ± 0.493 ^b^	153.182 ± 1.211

Note: Means with different superscripts (^A,B^ and ^a,b^ ) are significantly different (*p* < 0.01 and *p* < 0.05). All *p*-values were modified by Bonferroni correction.

**Table 6:** Associations of haplotype combination with body size traits in Qinchuan cattle.

Haplotype Combination	Body Size Traits (Mean ± SD)						
	Frequency	Body Length	Wither Height	Hip Height (cm)	Hip Width (cm)	Chest Circumference (cm)	Chest Depth (cm)
H1H1 (AA-AA-AA-AA-TT)	12% (50)	125.600 ± 1.128 ^B^	116.700 ± 0.711 ^A^	118.500 ± 0.596 ^a^	32.600 ± 0.778 ^B^	59.200 ± 0.998 ^A,b^	152.600 ± 2.243 ^B^
H2H3 (CC-GG-AC-GA-CC)	19% (80)	124.125 ± 0.892 ^B^	113.750 ± 0.562 ^B^	116.563 ± 0.471 ^B,b^	33.625 ± 0.615 ^B^	55.688 ± 0.789 ^B^	146.750 ± 1.773 ^B^
H3H3 (CC-GG-CC-GG-CC)	9% (40)	137.625 ± 1.262 ^A^	118.625 ± 0.795 ^A^	120.125 ± 0.667 ^A^	38.250 ± 0.870 ^A^	63.250 ± 1.115 ^A,a^	167.500 ± 2.507 ^A^

Note: Means with different superscripts (^A,B^ and ^a,b^ ) are significantly different (*p* < 0.01 and *p* < 0.05). All *p*-values were modified by Bonferroni correction.

**Table 7:** Sequences of primer pairs and amplification conditions for RT-qPCR.

Item	Function	Primer Sequences	Tm (^◦^C)	Production Size
STAT3	RT-PCR	F: 5-CACTTCTGCCAAGGGAGACT-3	59.5	261 bp
		R: 5-CGCGTATGCCCAATCTTGAC-3		
β-actin	Internal control	F: 5-CACCAACTGGGACGACAT-3	61	202 bp
		R: 5-ATACAGGGACAGCACAGC-3		
GAPDH	Internal control	F: 5-CCAACGTGTCTGTTGTGGAT-3	61	80 bp
		R: 5-CTGCTTCACCACCTTCTTGA-3		

## Abbreviations

CDD	Conserved Domains Database
FQC	fetal Qinchuan cattle
AQC	adult Qinchuan cattle
LD	linkage disequilibrium
MAS	marker-assisted selection
QTL	quantitative trait locus
HWE	Hardy-Weinberg equilibrium
